# Interactive Effects of Warming and Increased Precipitation on Community Structure and Composition in an Annual Forb Dominated Desert Steppe

**DOI:** 10.1371/journal.pone.0070114

**Published:** 2013-07-19

**Authors:** Yanhui Hou, Guangsheng Zhou, Zhenzhu Xu, Tao Liu, Xinshi Zhang

**Affiliations:** 1 State Key Laboratory of Vegetation and Environmental Change, Institute of Botany, Chinese Academy of Sciences, Beijing, China; 2 University of Chinese Academy of Sciences, Beijing, China; 3 Chinese Academy of Meteorological Sciences, Beijing, China; USDA-ARS, United States of America

## Abstract

To better understand how warming, increased precipitation and their interactions influence community structure and composition, a field experiment simulating hydrothermal interactions was conducted at an annual forb dominated desert steppe in northern China over 2 years. Increased precipitation increased species richness while warming significantly decreased species richness, and their effects were additive rather than interactive. Although interannual variations in weather conditions may have a major affect on plant community composition on short term experiments, warming and precipitation treatments affected individual species and functional group composition. Warming caused C_4_ grasses such as *Cleistogenes squarrosa* to increase while increased precipitation caused the proportions of non-perennial C_3_ plants like *Artemisia capillaris* to decrease and perennial C_4_ plants to increase.

## Introduction

Concurrent with increases in atmospheric CO_2_ and other greenhouse gases, global mean temperature has increased by 0.76°C since the 1850s and will further likely increase by 2.0–4.5°C by the end of this century [1]. Meanwhile, precipitation regimes are expected to be asymmetrically distributed, potentially causing mean global precipitation to increase by 7% [2]. Temperature and precipitation are important abiotic factors that directly and/or indirectly affect plant physiological processes and influence the growth, phenology, adaptive strategies, and productivity of individual plant species [3], [4]. Temperature and precipitation may also affect interactions among plants, their distributions, alter interspecific relationships [5]–[8], and often plant community structure and composition [9]–[11], ecosystem structure and function [12]–[17], and potentially feedbacks with climate change [18], [19]. Therefore, understanding the effect of hydrothermal changes on plant community structure and composition is crucial for evaluating the possible consequences of climate change on terrestrial ecosystems and may help inform regulatory policies to cope with climate change.

Evidence both from theoretical and empirical approaches has demonstrated that climatic changes, such as warming and precipitation change, profoundly affect plant community structure and composition [3], [8]–[11], [20]–[22]. Experimental warming can alter plant community structure and composition by altering competitive interactions and dominance hierarchies of different plant species or functional groups [6], [8], [9], [20], [23], [24]. For example, climate warming can increase the proportion of herbaceous plant species on arctic tundra [21], reduce the biomass of most species on a montane meadow [9], [25], or result in the loss of plant species [11], [26]. A variety of experiments have also examined plant community responses to changes in precipitation amounts [10], [27], [28], precipitation frequency [27], [29], [30], and precipitation seasonality [31], [32]. These studies provide abundant evidence showing climatic warming and changes in precipitation can strongly influence community structure and function.

However, most of these studies have focused on the effects of individual climate change drivers on plant communities, while the potential for additive or interactive effects of multiple environmental factors on plant communities remains unclear [28], [33]. The effects of multifactorial treatments differ from those of a single-factor treatment. For example, warming-induced reductions in soil water availability may limit positive effects of warming and increased precipitation on plant community and ecosystem processes [9], [34], [35]. Conversely, increased precipitation can decrease soil temperature and attenuate high-temperature stress induced by warming [36]. Meta-analyses have demonstrated the interactive effects of warming and increased precipitation on Net Primary Productivity (NPP) are small [37] or not significant [38]. This uncertainty highlights the importance of examining ecosystem responses to multiple abiotic factors [33]. But until now, few field experiments [9], [23], [33], [39]–[42] have factorially manipulated both temperature and precipitation.

The Intergovernmental Panel on Climate Change (IPCC) [1] reported that climate change will initially affect temperate steppe ecosystems because semiarid temperate steppe at high latitudes is one of the ecosystems which is most vulnerable to climate change [8], [20], [43]. Desert steppe located in northern China is an important part of the Eurasian grassland biome, which covers a total area of about 8.8 million ha, supports biodiversity, provides ecosystem services, and contributes to the socio-economic development of the region [44]. Temperatures in this region have increased substantially during the past 50 years [45] and are projected to experience “much greater than average” increases in the future [46]. Also, an increase in precipitation amount has been predicted in northern China [1]. A modeling study has shown temperature and precipitation are predicted to increase by 3°C and 30–100 mm, respectively [47]. Most research on climate change effects in the region has been on typical steppe [8], [41], [43], [45], [48]–[50]. Little attention has been paid to the response of desert steppe to climate change. Here our aim was to understand the impacts of climatic changes on desert steppe and dominant plant species.

To examine the interactive effects of temperature and precipitation on the plant community, we conducted a field experiment with warming manipulated by infrared heaters and increased precipitation simulated by watering at an annual forb dominated desert steppe in Damao County, Inner Mongolia since May 2011. Previous studies have demonstrated that water availability is a key factor regulating ecosystem responses to warming and increased precipitation in steppe system [43], [48]. Thus, changes in water availability resulting from warming and increased precipitation in the semiarid steppe will likely determine plant community composition. Given that increased precipitation improves soil water availability, we hypothesized that increased precipitation would promote both plant growth and species richness. Warming stimulates evapotranspiration and decreases soil water availability [9], [34], [48], exacerbating water stress, thus our second hypothesis was that warming would suppress plant growth and decrease species richness. Additionally, previous studies have shown additive effects of warming and increased precipitation [23], [48]. Third we hypothesized that warming and increased precipitation would independently affect plant community composition. Testing these hypotheses may provide new insights on the response of desert steppe to climate change.

## Materials and Methods

### Ethics Statement

All observational and field studies at the experimental site were undertaken with relevant permissions from the owners– Meteorological Bureau of Damao County, Inner Mongolia. The location is not privately-owned or protected in any way and the field studies did not involve endangered or protected species.

### Experimental Site

The experiment was conducted at a desert steppe (41°38′38.3″N, 110°19′53.3″E; 1409 m a.s.l.), in Damao County, Inner Mongolia, China. This site had not been grazed for 21 years. The region's typical continental climate is strongly influenced by the arid Mongolian airflow. The long-term (1978–2007) mean annual temperature was 4.6°C while the monthly mean temperature varied from –14.1°C in January to 21.4°C in July. Mean annual precipitation was about 255.2 mm with 67.6% distributed in the growing season (June to August). The soil at the experimental site is classified as chestnut according to Chinese classification, with mean bulk density of 1.23 g cm^–3^ and pH of 7.4. A calcium laminated layer lies at 20–30 cm depth below ground [51]. In generally, the desert steppe is dominated by a perennial C_3_ grass (*Stipa klemenzii*), an annual C_3_ forb (*Neopallasia pectinata*), an annual C_3_ sub-shrub (*Artemisia capillaris*) and a perennial C_4_ grass (*Cleistogenes squarrosa*). Our study site has a history of anthropogenic disturbance and is instead dominated by the annual forb (*Neopallasia pectinata*).

### Experimental Design

A randomized complete block design with two warming treatments (unwarmed [T0], warmed [T2]) and three watering treatments (ambient precipitation [W0], precipitation increased by 15% [W15], and precipitation increased by 30% [W30]) was manipulated since May 2011. The six treatment combinations were each replicated three times. Eighteen 2×2 m^2^ plots were arranged in 3×6 matrix (3 blocks with one of each treatment randomized within each block) with 1 m buffer separating adjacent plots. All the warmed plots were heated continuously (24 h/day, starting on 8 June 2011) by IR lamps (GHT220-800, Beijing Sanyuan Huahui Electric Light Source Co. Ltd., Chaoyang, Beijing, China) suspended 1.3 m above the ground. The lamp was 1.0 m long, 800 W at full electrical power fixed in a metal heater. A ‘dummy’ heater with the same shape and size as the infrared heater was mounted in the control plot to simulate the shading effects [34], [49]. A wooden square frame (2 m long and 0.2 m wide) was fastened to the ground along the sides of plot (0.1 m emerged aboveground and 0.1 m buried in soil) to prevent lateral movement of water and nutrients between individual plots and their surroundings. Based on the local long-term (1978–2007) monthly mean precipitation, watering volumes of each plot were 22.29 L, 39.41 L and 43.40 L in the W15 treatment plots and 44.14 L, 78.83 L and 86.36 L in the W30 treatment plots from June to August, respectively. Taking into account the frequency of precipitation in the growing season, we manipulated increased precipitation treatments once a week. All treatments were conducted only during the growing season each year.

### Meteorological Measurements

An air temperature and humidity monitoring instrument (HOBO Pro v2 Temp/RH, Onset Computer Corporation, Bourne, MA, USA) was mounted in a radiation shield at 40 cm above the ground in the center of each plot. Measurements taken every 2 seconds were averaged for each thirty minute period. In each plot, a thermocouple (HOBO S-TMB-M006, Onset Computer Corporation, Bourne, MA, USA) at 5 cm below the ground surface and a humidity transducer (HOBO S-SMA-M005, Onset Computer Corporation, Bourne, MA, USA) at 0–20 cm below the surface were installed to measure soil temperature and soil moisture content, respectively. Data were also recorded by an automatic data logger (HOBO H21-002, Onset Computer Corporation, Bourne, MA, USA) every 30 minutes. Meteorological data from June to August in 2011 and 2012 were obtained from an Automatic Meteorological Observation Instrument located 250 m northwest of the experimental site.

### Plant Community Characteristics

We measured plant community characteristics at the peak of plant biomass in late August in both years. A permanent 1×1 m^2^ quadrat was established at the center of each plot and vegetation characteristics in the quadrat were measured in 2011 and 2012. Plants were divided into different functional groups on the basis of life form (grasses, forbs, legumes, and sub-shrubs), photosynthetic pathway (C_3_ and C_4_ plants), and life history (annual/biennial and perennial plants) (Table 1). Ground coverage was visually estimated. A 1×1 m^2^ quadrat frame with 100 sub-grids (0.1×0.1 m^2^) was used to measure the coverage of each species. The coverages of different functional groups and the entire community were summed up by the species coverages. From the estimates of canopy coverage per species and functional group, we determined the relative coverage of each (i.e. percent cover). The individual numbers of each species and the plant community in the frame were counted. We determined the Species richness (*S*), Shannon-Wiener index (*H′*), and Pielou evenness index (*J′*) for each plot. The Pielou evenness index was calculated as: *J′* = *H′*/ln(*S*), where *H’* is the Shannon-Wiener index and calculated as: *H′ = *–∑*Pi*ln(*Pi*), where *Pi* is the proportional number of species *i* and *S* is the species richness in the community.

**Table 1:** The plant species, life form (LF), photosynthetic pathway (PP), life history traits (LH) and the percent cover of plant species within the quadrats (1 × 1 m^2^).

Species	LF	PP	LH	Percent cover (%)	
				2011	2012
*Cleistogenes squarrosa*	Grass	C_4_	Perennial	12.27	13.74
*Stipa klemenzii*	Grass	C_3_	Perennial	6.31	7.23
*Tribulus terrestris*	Forb	C_4_	Annual	0.08	2.38
*Chenopodium glaucum*	Forb	C_4_	Annual	/	0.95
*Neopallasia pectinata*	Forb	C_3_	Annual	49.78	14.77
*Erodium stephanianum*	Forb	C_3_	Annual	4.67	0.48
*Heteropappus altaicus*	Forb	C_3_	Perennial	2.33	2.46
*Convolvulus ammannii*	Forb	C_3_	Perennial	0.26	0.08
*Allium bidentatum*	Forb	C_3_	Perennial	0.52	0.40
*Ixeris chinensis*	Forb	C_3_	Perennial	/	0.32
*Salsola collina*	Forb	C_4_	Annual	2.25	2.38
*Iris tenuifolia*	Forb	C_3_	Perennial	/	0.24
*Scorzonera divaricata*	Forb	C_3_	Perennial	0.09	0.16
*Astragalus galactites*	Legume	C_3_	Perennial	9.68	2.54
*Lagochilus ilicifolius*	Sub-shrub	C_3_	Perennial	0.69	0.16
*Artemisia capillaries*	Sub-shrub	C_3_	Annual	10.29	51.07
*Caragana stenophylla*	Sub-shrub	C_3_	Perennial	0.78	0.64

### Statistical Analysis

Three-way ANOVAs were carried out to test the main and interactive effects of warming, increased precipitation, and year on community composition (the percent cover of individual species and functional groups), and community structure (species richness, Shannon diversity, and Pielou evenness). One-way ANOVA with a Duncan’s multiple range tests was used to test the statistical significance in the mean values of the treatments. Statistical significances of all tests were set at *P*<0.05. All statistical analyses were conducted with SPSS 17.0 software (SPSS Institute Incorporated, Chicago, Illinois, USA), and all the data were normally distributed as determined by the Shapiro-Wilk *W* statistic [52] prior to statistical analysis.

## Results

### Environmental Factors

**Figure 1:**
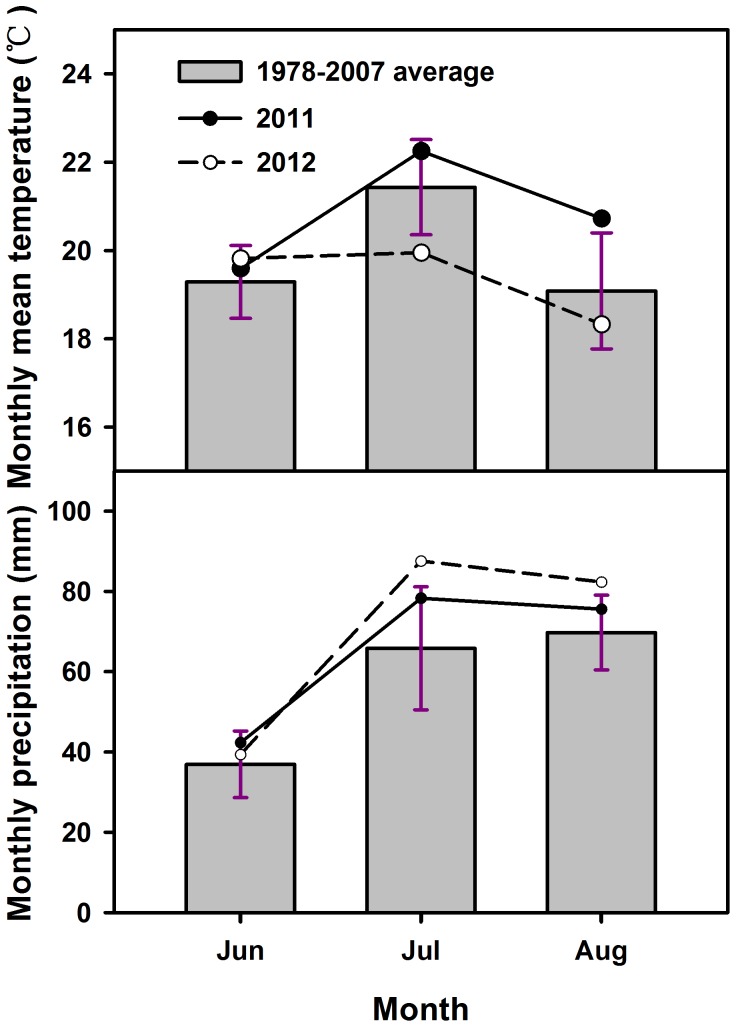
Monthly average temperature (top) and total monthly precipitation (bottom) in the growing season. Gray bars indicate the regional monthly averages (mean ± SE) from 1978 to 2007.

Compared to the 30-year averages, weather conditions at our experimental site were unusually hot and wet and unusually cool and wet in the 2011 and 2012 growing seasons, respectively (Fig. 1).

**Figure 2:**
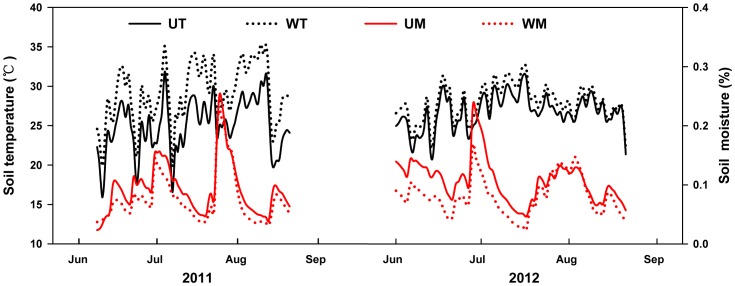
Daily mean soil temperature and soil moisture during the growing season in 2011 and 2012. UT, soil temperature in unwarmed plot; WT, soil temperature in warmed plot; UM, soil moisture in unwarmed plot; WM, soil moisture in warmed plot.

During the two growing season periods, the soil temperature showed strong interannual variation at 5 cm deep (*P*<0.01; Fig. 2). The soil temperatures in the warmed and control plots in 2011 were 5.18°C and 2.66°C higher than those in 2012, respectively. Warming induced by infrared heaters elevated the mean soil temperature by 4.10°C (2011, *P*<0.01) and 1.58°C (2012, *P*<0.01), but reduced the mean soil moisture (0–20 cm, v/v) by 1.66% (2011, *P*<0.05) and 2.63% (2012, *P*<0.01) (Fig. 2).

### Percent Cover of Dominant Species

**Figure 3:**
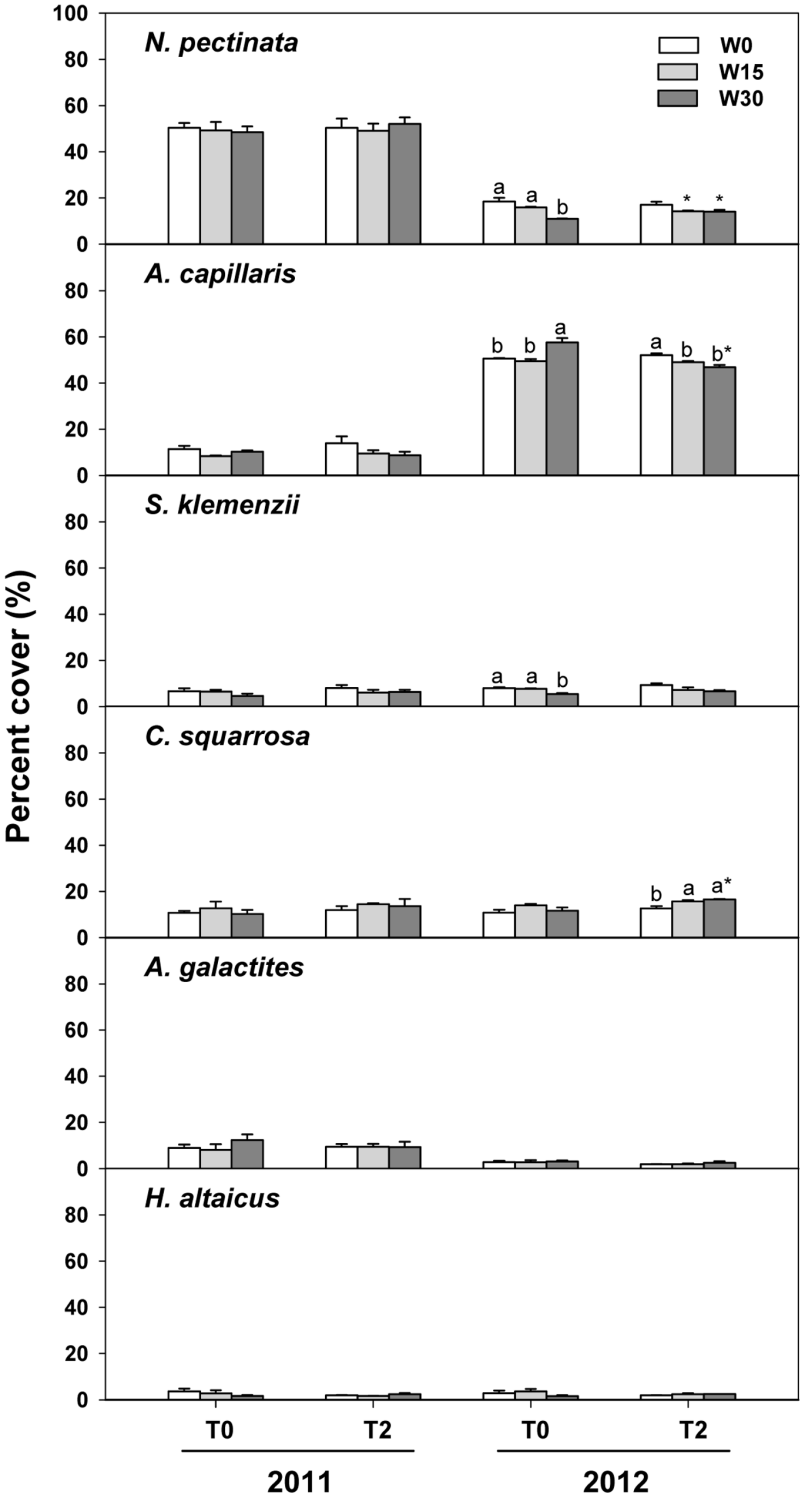
Effects of warming and increased precipitation on the percent cover of dominant species in 2011 and 2012. *N. pectinata*, *Neopallasia pectinata*; *A. capillaris*, *Artemisia capillaris*; *S*. *klemenzii*, *Stipa klemenzii*; *C*. *squarrosa*, *Cleistogenes squarrosa*, *H*. *altaicus*, *Heteropappus altaicus*; *A*. *galactites*, *Astragalus galactites*. Values are the means ± SE of three replications. Different lowercases indicate significant difference among different precipitation treatments in the same temperature treatment within the same year (p<0.05); * indicates significant difference between unwarmed and warmed treatments in the same precipitation treatment within the same year (p<0.05).

Warming significantly increased the percent cover of *C. squarrosa* (*F*_1, 24_ = 7.122, *P*<0.05) while it had no significant effects on other species (Table 2; Fig. 3). Increased precipitation had significant impacts on the percent cover of *A. capillaris* (*F*_2, 24_ = 4.718, *P*<0.05) and *S. klemenzii* (*F*_2, 24_ = 6.192, *P*<0.01). Increased precipitation (W15 vs. W0) significantly decreased the percent cover of *A. capillaris* by 9.07% (*P*<0.01), and increased precipitation (W15 and W30 vs. W0) also reduced the percent cover of *S. klemenzii* by 13.75% (*P* = 0.095) and 27.83% (*P*<0.01), respectively.

**Table 2:** Results (*P*-values) of three-way ANOVAs on the effects of temperature, precipitation, year and their interactions on the percent cover of dominant species.

Sources of Variation	df	*N. pectinata*	*A. capillaris*	*S*. *klemenzii*	*C*. *squarrosa*	*A. galactites*	*H. altaicus*
T	1	0.668	0.124	0.135	**0.013**	0.476	0.200
P	2	0.259	**0.019**	**0.007**	0.075	0.441	0.495
Y	1	**<0.001**	**<0.001**	0.062	0.179	**<0.001**	0.802
T×P	2	0.357	**0.001**	0.265	0.459	0.570	0.083
T×Y	1	0.677	**0.020**	0.814	0.735	0.783	0.742
P×Y	2	0.292	0.121	0.811	0.731	0.738	0.524
T×P×Y	2	0.984	0.073	0.977	0.937	0.485	0.898

Abbreviations: T, temperature; P, precipitation; Y, year; *N. pectinata*, *Neopallasia pectinata*; *A. capillaris*, *Artemisia capillaris*; *S. klemenzii*, *Stipa klemenzii*; *C. squarrosa*, *Cleistogenes squarrosa*; *H. altaicus*, *Heteropappus altaicus*; *A. galactites*, Astragalus galactites. Significant level: at <0.05 in bold.

We observed shifts in species relative contributions to the plant community between years (Table 2; Fig. 3). The percent cover of *A. capillaris* increased from 10.29% in 2011 to 51.07% in 2012 (*F*_1, 24_ = 2722.381, *P*<0.001), while the percent cover of *N. pectinata* (from 49.78% to 14.77%; *F*_1, 24_ = 677.013, *P*<0.001) and *A. galactites* (from 9.68% to 2.54%; *F*_1, 24_ = 71.925, *P*<0.001) decreased during the same period (Table 1 & 2; Fig. 3). We failed to detect significant differences in the percent cover of *S. klemenzii* (*F*_1, 24_ = 3.847, *P = *0.062), *C. squarrosa* (*F*_1, 24_ = 1.914, *P = *0.179), and *H. altaicus* (*F*_1, 24_ = 0.064, *P = *0.802) between years.

We detected an interactive effect of warming and water addition treatments on the percent cover of only one plant species, *A. capillaris* (*F*_2, 24_ = 10.155, *P*<0.01). In addition, the effects of warming on the percent cover of *A. capillaris* varied with year (*F*_1, 24_ = 6.254, *P*<0.05; Table 2). Warming increased the percent cover of *A. capillaris* by 7.02% in 2011 while decreased that by 6.05% in 2012. We did not observe any interactions among warming, increased precipitation, and year on the percent cover of other dominant species (Table 2).

### Percent Cover of Functional Groups

**Figure 4:**
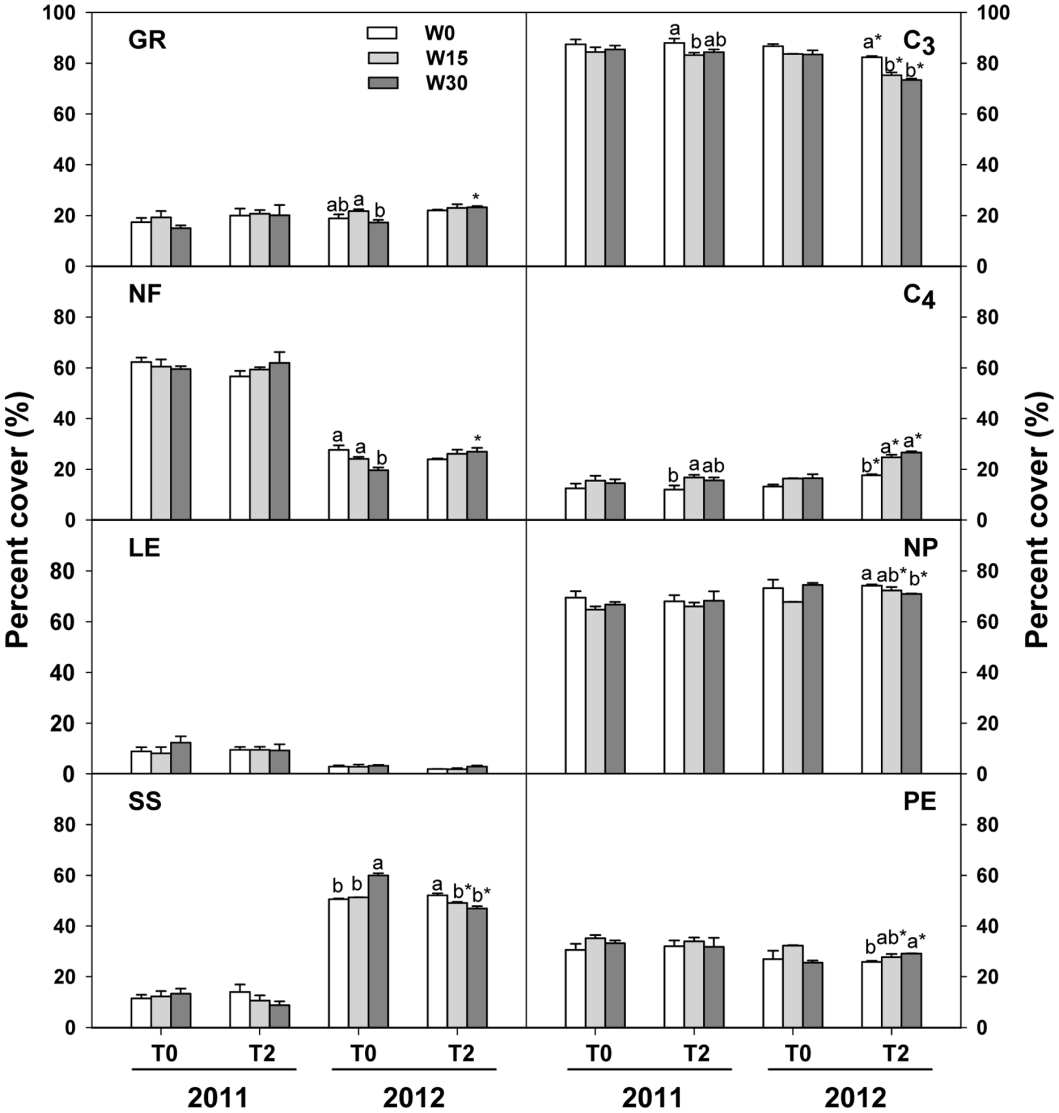
Effects of warming and increased precipitation on the percent cover of different functional groups in 2011 and 2012. GR, grasses; NF, nongraminous forbs; LE, legumes; SS, sub-shrubs; C_3_, C_3_ plants; C_4_, C_4_ plants; NP, non-perennial (annual/biennial) plants; PE, perennial plants. Values are the means ± SE of three replications. Different lowercases indicate significant difference among different precipitation treatments in the same temperature treatment within the same year (p<0.05); * indicates significant difference between unwarmed and warmed treatments in the same precipitation treatment within the same year (p<0.05).

Experimental warming increased the percent cover of grasses by 17.8% (*F*_1, 24_ = 8.912, *P*<0.01) and decreased the percent cover of sub-shrubs by 8.8% (*F*_1, 24_ = 10.753, *P*<0.01) while it had no effect on that of forbs (*F*_1, 24_ = 0.032, *P = *0.859) or legumes (*F*_1, 24_ = 0.418, *P = *0.524; Table 3). Increased precipitation did not affect any life form functional groups. In contrast, there were significant shifts in functional groups between years (Table 3; Fig. 4). For example, we observed several functional groups increasing (grasses, *P = *0.046; sub-shrubs, *P*<0.001) from 2011 to 2012 while forbs (*P*<0.001) and legumes (*P*<0.001) decreased during the same period. The relative contributions of forbs (*F*_2, 24_ = 5.995, *P*<0.01) and sub-shrubs (*F*_2, 24_ = 12.708, *P*<0.001) to community cover were both significantly affected by the interactions of warming and increased precipitation (Table 3). Increased precipitation (W15 and W30 vs. W0) decreased the percent cover of forbs by 5.99% and 12.08% in unwarmed plots while increased that by 6.14% and 10.51% in warmed plots. In contrast, increased precipitation (W15 and W30 vs. W0) enhanced the percent cover of sub-shrubs by 2.53% and 18.17% in unwarmed plots while increased that by 9.86% and 15.85% in warmed plots (Fig. 4).

**Table 3:** Results (*P*-values) of three-way ANOVAs on the effects of temperature, precipitation, year and their interactions on the percent cover of different functional groups.

Sources of Variation	df	GR	NF	LE	SS	C_3_	C_4_	NP	PE
T	1	**0.006**	0.859	0.524	**0.003**	**<0.001**	**<0.001**	0.612	0.612
P	2	0.230	0.901	0.376	0.377	**<0.001**	**<0.001**	**0.047**	**0.047**
Y	1	**0.046**	**<0.001**	**<0.001**	**<0.001**	**<0.001**	**<0.001**	**<0.001**	**<0.001**
T×P	2	0.298	**0.008**	0.633	**<0.001**	0.120	0.120	0.332	0.332
T×Y	1	0.862	0.158	0.846	0.070	**<0.001**	**<0.001**	0.911	0.911
P×Y	2	0.933	0.396	0.797	0.176	0.204	0.204	0.977	0.977
T×P×Y	2	0.975	0.877	0.416	0.144	0.545	0.545	0.256	0.256

Abbreviations: T, temperature; P, precipitation; Y, year; GR, grasses; NF, nongraminous forbs; LE, legumes; SS, sub-shrubs; C_3_, C_3_ plants; C_4_, C_4_ plants; NP, non-perennial (annual/biennial) plants; PE, perennial plants. Significance level: *P*<0.05 in bold.

The percent cover of C_3_ and C_4_ plants were both significantly affected by temperature (*F*_1, 24_ = 31.513, *P*<0.001), precipitation (*F*_2, 24_ = 16.796, *P*<0.001), and year (*F*_1, 24_ = 40.592, *P*<0.001), but the warming effects varied by year (*F*_1, 24_ = 22.762, *P*<0.001; Table 3). Increased precipitation negatively affected the percent cover of C_3_ plants while increased the relative contributions of C_4_ plants. In 2011, there was no significant warming effect on C_3_ plants or C_4_ plants. But in 2012, warming significantly decreased the percent cover of C_3_ plants by 9.56% (*P*<0.01) but increased that of C_4_ plants by 54.91% (*P*<0.01; Fig. 4).

Neither perennial plants nor non-perennial plants differed between the two temperature treatments (*F*_1, 24_ = 0.265, *P = *0.612; Table 3). In contrast, increased precipitation (*F*_2, 24_ = 3.492, *P*<0.05) had significant effects on perennial plants and non-perennial plants. Increased precipitation (W15 vs. W0) elevated the percent cover of perennial plants by 12.21% (*P*<0.05) while increased that of non-perennial plants by 4.94% (*P*<0.05; Fig. 4). We observed differences between years for perennial and non-perennial plants (*F*_1, 24_ = 19.647, *P*<0.001). The percent cover of perennial plants decreased from 32.79% in 2011 to 27.87% in 2012 (*P*<0.01), while the percent cover of non-perennial plants increased from 67.21% in 2011 to 72.13% in 2012 (*P*<0.01). We failed to detect any interactions among warming, increased precipitation, and year on perennial plants or non-perennial plants (Table 3).

### Plant Community Diversity

**Figure 5:**
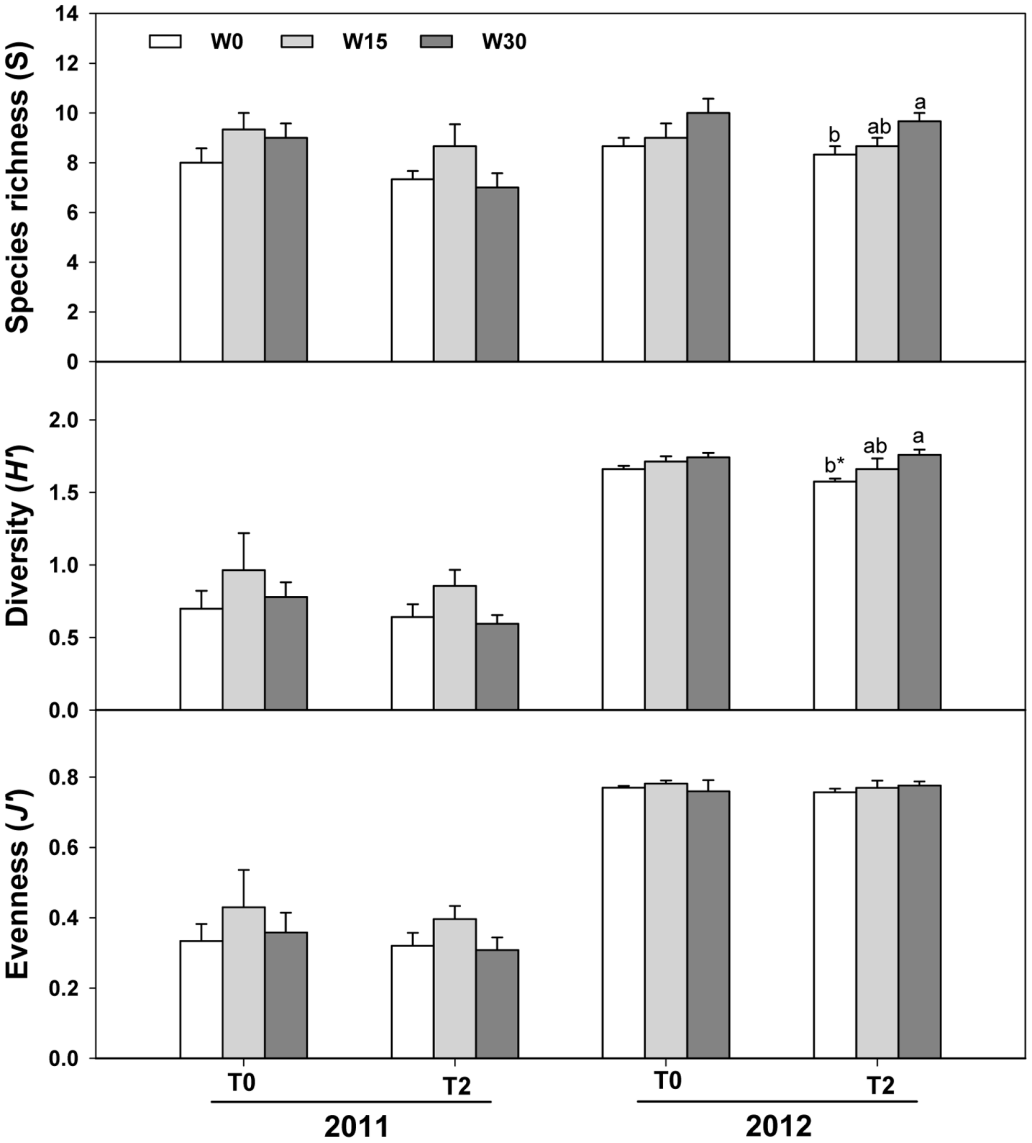
Effects of warming and increased precipitation on species richness (*S*) of the community, Shannon-Wiener index (*H’*), and Pielou evenness (*J’*) (mean ± SE) in 2011 and 2012. T0, unwarmed; T2, warmed; W0, ambient precipitation; W15, precipitation increased by 15%; W30, precipitation increased by 30%. Different lowercases indicate significant difference among different precipitation treatments in the same temperature treatment within the same year (p<0.05); * indicates significant difference between unwarmed and warmed treatments in the same precipitation treatment within the same year (p<0.05).

Experimental warming reduced species richness by 8.72%, but it did not influence *H’* or *J’* across the two years. Increased precipitation also had no effect on *H’* or *J’* across the two years, but increased species richness (Table 4; Fig. 5). In 2011, warming reduced species richness by 14.49% while increased precipitation had no significant effect on species richness. In 2012, species richness was increased as precipitation increased and there was a significant difference between W0 treatment andW30 treatment (Fig. 5).

**Table 4:** Effects (*P*-values) of temperature, precipitation, year and their interactions on plant species richness, Shannon diversity and Pielou evenness by three-way ANOVAs.

Source of variation	df	*S*	*H’*	*J’*
T	1	**0.028**	0.192	0.487
P	2	**0.044**	0.118	0.232
Y	1	**0.013**	**<0.001**	**<0.001**
T×P	2	0.603	0.997	0.986
T×Y	1	0.221	0.524	0.564
P×Y	2	**0.047**	0.152	0.393
T×P×Y	2	0.603	0.727	0.862

Abbreviations: T, temperature; P, precipitation; Y, year; *S*, species richness; *H’*, Shannon diversity; *J’*, Pielou evenness. Significant *P*-values at 0.05 level are bolded.

In the annual forb dominated community, diversity measures (*S*, 10.22%, *P*<0.05; *H’*, 122.37%, *P*<0.01; and *J’*, 113.89%, *P*<0.01) were greater in 2012 than 2011.We failed to detect any interactions among warming, increased precipitation, and year on *H’* or *J’* across the two years (Table 4).

## Discussion

### Combined Effects of Weather Condition and Simulated Hydrothermal Treatments on Community Dynamics

In semiarid steppe, precipitation is the main factor limiting plant growth [32], [48]. During the experiment, ambient precipitation was larger than the 30 year mean precipitation, which resulted in more productivity and enormous interannual variations of measured variables during the two years’ experiment was maintained. Interannual variability of community structure and function affected by annual weather conditions have been reported across different regions [53], [54]. Also, weather conditions often interact with hydrothermal treatments in experiments affecting community dynamics [40], [55], [56], sometimes eclipsing treatment effects to a certain extent [42], [57]. We documented significant interannual variability in measured abiotic response variables (e.g. soil temperature and moisture) illustrating that the effects of weather conditions can be enormous and may exceed treatment effects. Since our water additions occurred during a sequence of years with greater than average precipitation, effects of water additions are likely to be conservative.

In addition to precipitation amount, precipitation frequency and intensity are also important for plant growth and should be taken into account under future climate change scenarios [1], [58]. But in this study, artificial precipitation frequency was once a week to simplify the treatment. Precipitation intensity was artificially changed once a month to match monthly historic averages, which would affect plant growth and community structure and composition. A limit to this type of experimental manipulation, however, is that water additions on a sunny day (low humidity, high winds, higher evapotranspiration) may be effectively less than a same addition during a natural rain event when humidity is likely higher and evapotranspiration is likely lower.

### Effects of Warming and Increased Precipitation on Community Structure

Experimental warming decreased species richness primarily by reducing the prevalence of annual forb species. Other climate change experiments have also detected reductions in richness [11], [57], [59], but others observed no effects on species richness [55], [56]. Klein *et al*. [11] attributed species loss induced by warming to heat stress and litter accumulation. Yang *et al*. [41] concluded that warming indirectly affected species richness by altering soil water availability. In this region, species loss is expected to be the result of direct and indirect effects of warming. For instance, some psychrophilic C_3_ plants (e.g. *Allium bidentatum*) would disappear as the result of heat stress. Moreover, they are affected by reduction of soil water availability owing to warming. Since the lost species persist in seed bank and will germinate in plots with added soil moisture, such effects are likely ephemeral and should be examined in long-term.

Unlike the negative effects of warming on species richness, increased precipitation enhanced species richness in this study (Fig. 5). For instance, the annual C_4_ forb- *Chenopodium glaucum* appeared in wetter plots. This finding supports the results reported in other grassland ecosystems [10], [41], [55]. Increased precipitation may directly affect species richness through altering soil moisture and can relieve warming-induced heat stress by reducing soil temperature. Thus, increased precipitation plays a positive role in maintaining the stability of grassland ecosystem.

Neither warming nor increased precipitation had significant effects on the diversity index and evenness, while strong interannual variations were detected in this study (Table 4; Fig. 5). Our two year experiment is a relatively short duration which may be insufficient for detecting changes in many species, especially perennial plants. Thus, we recommend caution in interpreting the effects of short-term weather manipulations as an indicator of the effects of long-term climatic shifts. Also, treatment effects on species diversity might be under-estimated or over-estimated because treatment plots are typically small and cannot involve all species or eliminate the effects of surrounding species. Overall, hydrothermal treatment effects on community structure are small and may be difficult to predict from field experiments at small spatial and temporal scales [42], [60]. Since interannual variation in weather is so extreme, experimental design may require manipulations over a decade or more to reveal the consequences of climate change.

### Effects of Warming and Increased Precipitation on Functional Groups

Because of their intrinsic hydrothermal sensitivity, functional groups can show different speeds and amplitudes of their responses to environmental change which results in shifts in their competitive abilities and dominance hierarchies among individual species or functional groups [4], [9], [41], [42]. This may also affect community structure and composition. In our experiment, the community was dominated by forbs, C_3_ plants, and non-perennial plants in 2011. But by 2012, the dominant group was replaced by sub-shrubs and the relative contributions of C_4_ plants and non-perennial plants were significantly elevated (Fig. 4). The expansion of sub-shrubs in 2012 mainly resulted from overgrowth of *A. capillaris*, whose percent coverage increased nearly four times relative to that in 2011 (Fig. 3). The increased percent coverage of *C. squarrosa* resulted in the enhancement of C_4_ species in 2012.

The result showing that experimental warming and increased precipitation both significantly affect community composition confirms observations in other grassland communities [57], [61], [62]. Annual or biennial plants respond rapidly to environmental change. In this study, however, increased precipitation decreased the percent cover of non-perennial plants, which may be due to the considerable growth of perennial C_4_ plants (e.g. *C. squarrosa*) under increasing moisture conditions. The ability of C_4_ plants to flourish relative to C_3_ species in warmed and increased precipitation plots lends support to the conclusion that C_4_ plants have a competitive advantage in warmer and wetter climate scenarios [13], [42]. This was similar to results in other grassland ecosystems [4], [42], [63]. Such changes in plant functional groups may alter the response characteristics of communities and ecosystems to future climate change [64].

### Effects of Warming and Increased Precipitation on Dominant Species

Dominant species, as the most prevalent species in the community, strongly affect biotic conditions and are often key drivers of community dynamics [6]. Responses of dominant species to environmental factors sometimes mirror the entire community [23]. For instance, *N. pectinata* and *A. capillaris* dominated our community in 2011 and 2012, respectively (Fig. 1 & 3), and their responses to hydrothermal changes are consistent with community dynamics. As annual plants, *N. pectinata* and *A. capillaris* effectively sensed and responded to hydrothermal changes, and they could take full advantage of scarce resources (such as water) to quickly grow and reproduce which allowed them to dominate the community.

In ecosystems with high environmental stress, environmental conditions are the primary limiting factors [65]. Because of their different competitive abilities between species, weather conditions and hydrothermal treatments had different impacts on species, which influenced the composition of functional groups and community. When other resources are not limiting, warming often promotes plant growth by stimulating metabolism and enhancing photosynthetic rates. But in this study, most species grew well when precipitation increased and less in warmed plots (Fig. 3). That may be because changes in soil water availability induced by warming can offset the positive warming effects in this semiarid region where precipitation is often the most limiting factor. Increased precipitation promoted the growth of *A. capillaris* and *S. klemenzii* but decreased their relative contributions to plant community, which may be caused by growth of other species.

### Conclusions

In this study, plant community composition in the desert steppe was altered under simulated climatic changes. The effects of increased precipitation influenced species-, functional group-, and community-level responses more than the effects of warming but variation in weather condition had a greater effect than either treatment. These results demonstrate that future climatic warming if coupled with added moisture may not have drastic negative effects on community structure. Overall, soil moisture was the dominant factor affecting species richness, the percent cover of species and functional groups. Significant differences in percent cover among individual species and functional groups under different hydrothermal treatments were observed. Increased precipitation promoted the growth of C_4_ plants such as *C. squarrosa* but decreased the percent cover of annual Artemisia plants like *N. pectinata* and *A. capillaris*, while warming caused C_4_ grasses to increase and C_3_ sub-shrubs to decrease; and their effects on plant community composition were additive rather than interactive.
